# Recent advances in oesophageal diseases

**Published:** 2014

**Authors:** David Al Dulaimi

**Affiliations:** Department of Gastroenterology, Alexandra Hospital, Redditch, UK

## Abstract

Dong Y, Qi B, Feng XY, Jiang CM. **Meta-analysis of Barrett's esophagus in China.** World J Gastroenterol 2013;19(46):8770-8779

The disease pattern of Barrett's esophagus (BE) in China is poorly characterised particularly in comparison with other developed countries. This meta-analysis of 3873 cases of BE collated from 69 clinical studies conducted in 25 provinces between 2000 and 2011 investigated the epidemiology and characteristics of BE in China compared to Western countries. The total endoscopic detection rate of BE was 1.0% (95%CI: 0.1%-1.8%) with an average patient age of 49.07 ± 5.09 years, lower than many Western countries.The authors postulate this may be attributed to environmental risk factor variation, distinct genetics and different medical practice including diagnostic criteria for BE and expertise in endoscopy. This study identified a 1.781 male predominancefor BE in China, consistent with Western reports. Short-segment BE accounted for 80.3% of cases with island type and cardiac type the most common endoscopic (44.8%) and histological (40.0%) manifestations respectively. Of the 1283 BE cases followed up for three to 36 months the incidence of esophageal cancer was 1.418 per 1000 person-years, lower than the incidence reported in Western countries.

Lee HS, Jeon SW. **Barrett esophagus in Asia: same disease with different pattern. **ClinEndosc 2014;47(1):15-22

Barrett's esophagus (BE) is a common, pre-cancerous condition characterised by intestinal metaplasia of squamous esophageal epithelium usually attributed to chronic gastric acid exposure. This review article explores important differences in the disease pattern of BE between Asian and the Western countries.

Overall the prevalence of BE is lower in Asia compared to the West with a greater proportion of short-segment type. The authors identify great variability in the endoscopic and pathologic diagnostic criteria for BE. Many of the studies in Asian countries did not use a standardised four-quadrant biopsy protocol which may have led to an underestimation of BE prevalence. The review highlights an increasing incidence of esophageal adenocarcinoma in the West but unclear disease trend in Asia with inter-country variability. Similarly in Asian and Western countries BE is associated with the presence of hiatus hernia, advancing age, male gender, alcohol consumption, smoking, abdominal obesity and longer duration of gastro-esophageal reflux disease. The authors postulate that *Helicobacter pylori* infection, more prevalent in Asia than the West, may have a protective effect on BE.

There is a need for larger, prospective studies to further clarify the disease pattern of BE in Asian countries. Clearly standardisation of the diagnostic process for BE is important to validate the differences in disease trends between Asian and Western countries.

Kiadaliri AA. **Gender and social disparities in esophagus cancer incidence in Iran, 2003-2009: a time trend province-level study.**Asian Pac J Cancer Prev 2014;15(2):623-7

Esophageal cancer (EC) is a major cause of morbidity and mortality particuarly in Iran where the incidence rate exceeds the global average. An understanding of the factors influencing the province-specific incidence of EC in Iran is important to inform disease-prevention strategies and address health inequalities. This ecological study used cancer registry data to investigate the relationship between gender and social class and the incidence of EC in Iran at province-level between 2003 and 2009. The age standardised incidence rates (ASIR) of EC were greatest in the Northern provinces of Iran, specifically Razavi Khorasan in males and Kordestan in females. Overall the EC incidence did not significantly differ according to gender.

Interestingly, during the study period the ASIR increased by 4.6% per year in females (p=0.08) and 6.5% per year in males (p=0.02). This may reflect increasing rates of establised risk factors for EC including obsesity and gastro-esophageal reflux disease alongside more vigilant recording of new cases. Social class was inversely associated with the ASIR of EC regardless of gender which may be attributed to class differences in risk factor distribution particularly smoking, diet and obesity. An appreciation for the limitations of an epidemiological study is important when interpreting results which should be further evaluated in future studies.

Islami F et al.**Determinants of gastroesophageal reflux disease, including hookah smoking and opium use- A cross-sectional analysis of 50,000 individuals. **PLoS One 2014;9(2):e89256

Gastroesophageal reflux disease (GERD) is a highly prevalent cause of gastrointestinal symptoms worldwide incurring great cost to the primary and secondary healthcare sectors. An improved understanding of the factors which influence GERD symptoms in low- to medium- income countries may inform public health initiatives. This study analysed prospective data from the Golestan cohort study, primarily established to investigate determinants of upper gastrointestinal cancers, toexplore the risk factors influencing GERD symptoms (regurgitation and/or heartburn) in 50,045 individuals aged 40-75 years in Golestan Province, Iran enrolled between 01/2004 and 06/2008.Of note, 39.12% of individuals denied ever experiencing GERD symptoms. A further 19.89% reported at least once weekly GERD symptoms with 11.83% experiencing daily symptoms. Severe symptoms, defined as disturbing daily work or sleep, were recorded by 11.33% of individuals.

Separately the occurrence of daily GERD symptoms and severe symptoms were inversely associated with male gender (OR 0.36, 95% CI 0.33-0.39 both), level of formal education (p=0.01 and p=0.001 respectively), wealth score (p<0.001 both) and regular nass chewing (OR 0.86, 95% CI 0.75-0.98 and OR 0.87, 95% CI 0.76-0.99 respectively)and were positively associated with body mass index (p<0.001 both), intensity of physical activity (p=0.04 both), cigarette pack years (p<0.001 both), alcohol consumption (OR 1.36, 95% CI 1.13-1.64 and OR 1.53, 95% CI 1.28-1.83 respectively) and opium use (OR 1.82, 95% CI 1.67-1.99 and OR 1.70, 95% CI 1.55-1.87 respectively).In addition hookah smoking had a borderline significant correlation with mild and moderate severity GERD symptoms in individuals who had never smoked cigarettes (OR 1.41, 95% CI 1.00-1.99 and OR 1.25, 95% CI 0.99-1.57 respectively).

Overall this large study contributes useful data to inform the prevention and management of GERD symptoms particularly regarding the use of hookah, opium and nass which was previously unclear.

Barbera M et al.** The human squamous oesophagus has widespread capacity for clonal expansion from cells at diverse stages of differentiation. **Gut 2014;0:1–9. doi:10.1136/gutjnl-2013-306171

Current knowledge on human esophageal tissue homeostasis and injury repair is derived predominantly from murine models and hence may be inaccurate due to cellular and architectural differences. This study used 3D imaging in conjunction withstaining for cell lineage markers to investigate the cellular mechanisms involved in homeostasis of the normal human squamous esophagus in 10 participants undergoing esophagectomy for esophageal cancer. The self-renewal potential of cell subpopulations was also assessed using in vitro and in vivo assays.

A decreasing gradient of cell proliferation was observed from the inter-papillary basal layer to the tip of the papilla where there was no evidence of mitosis. The expression ofβ1-integrin, a putative stem cell marker, was consistent throughout the basal layer and therefore the entire basal layer can be considered undifferentiated. Quiescent β1-integrin/CD34-positive cells which failed to stain for CD45, S-100 or F4-80were identified at the tip of the papilla suggesting this is an extension of the basal layer. Contrary to previous data, this study found progenitor cells widely distributed in human esophageal tissue and included already differentiated epithelial cells. This insight into esophageal homeostasis may inform future studies exploring the pathological mechanisms underpinning homeostatic disruption in disease states such as Barrett's esophagus.

Papers were prepared by:

**Drs Ishfaq Ahmad **and** Luke Materacki, **Department of Medicine, Alexandra Hospital, Redditch, UK

